# Spatial Immune Coding in Tumor-Draining Lymph Nodes: Functional Compartmentalization of Immune Activation and Immunosuppression

**DOI:** 10.3390/ijms27167113

**Published:** 2026-08-08

**Authors:** Jinjie Li, Wei Ping, Ruijie Zhang, Lin Peng, Yujie Zhang, Li Zhang

**Affiliations:** 1 Department of Oncology, Tongji Hospital, Tongji Medical College, Huazhong University of Science and Technology, Wuhan 430030, China; 13461919752@163.com (J.L.); 13996484451@163.com (L.P.); tjzyj@tjh.tjmu.edu.cn (Y.Z.); 2 Department of Thoracic Surgery, Tongji Hospital, Tongji Medical College, Huazhong University of Science and Technology, Wuhan 430030, China; pingwei@tjh.tjmu.edu.cn (W.P.); lmzrj@163.com (R.Z.)

**Keywords:** tumor-draining lymph nodes, spatial multi-omics, spatial transcriptomics, spatial proteomics, functional compartmentalization, immune activation, immunosuppressive niche, tertiary lymphoid structures

## Abstract

Tumor-draining lymph nodes (TDLNs) are important immune organs linking primary tumors with systemic immune responses. They support tumor antigen presentation, T-cell priming, and effector immune responses, but under sustained tumor influence they can also be remodeled into microenvironments that promote immune escape and metastatic colonization. Conventional methods, including flow cytometry, bulk RNA sequencing, and routine immunohistochemistry, have advanced our understanding of TDLN immunity but cannot simultaneously preserve tissue architecture, cell identity, and spatial cell–cell relationships. Recent advances in spatial transcriptomics, spatial proteomics, and multiplexed imaging enable in situ analysis of immune cells, stromal cells, vascular structures, and their interactions within TDLNs. Emerging evidence suggests that TDLNs are not immunologically homogeneous organs, but gradually develop spatially distinct immune-activation and immunosuppressive regions during tumor progression. Activation regions are associated with HEV-mediated lymphocyte entry, DC–T-cell priming, B-cell follicles, and germinal-center reactions, whereas suppressive regions are enriched in Treg cells, exhausted T cells, suppressive myeloid cells, tumor-reprogrammed FRCs, and myeloid–CAF niches. This review summarizes spatial multi-omics studies of TDLN functional compartmentalization and discusses the potential value of TDLN spatial immune states in predicting immunotherapy response, assessing metastatic risk, and guiding precision treatment, thereby providing a reference for future spatial multi-omics studies of TDLNs.

## 1. Introduction

**Figure 1:**
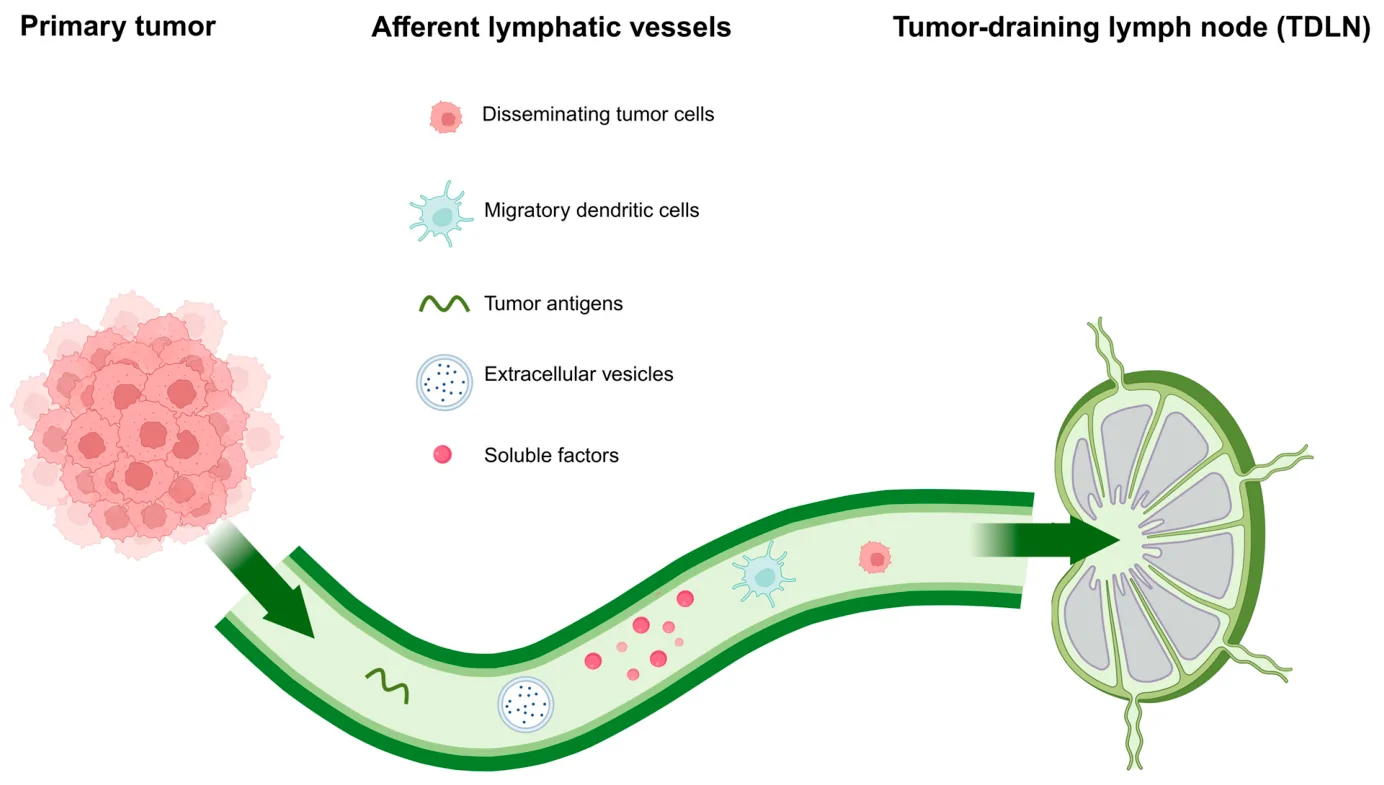
Anatomical and functional linkage between primary tumors and tumor-draining lymph nodes (TDLNs). Tumor-derived signals, including antigens, soluble mediators, extracellular vesicles, migratory dendritic cells, and disseminating tumor cells, are transported from primary tumors to TDLNs through afferent lymphatic vessels. These inputs shape the immune organization and functional state of TDLNs, which can support either antitumor immune activation or tumor-associated immune remodeling.

Tumor-draining lymph nodes (TDLNs) are regional lymph nodes anatomically linked to the tumor microenvironment (TME) through a unidirectional lymphatic network. They are often the first lymphoid sites encountered during systemic dissemination of cancer cells. TDLNs have a paradoxical role: they can initiate host antitumor immunity, but may also facilitate tumor spread [1]. The anatomical and functional connection between primary tumors and TDLNs through afferent lymphatic vessels is illustrated in Figure 1. On the one hand, TDLNs are central sites for initiating and sustaining antitumor immune responses. They contain abundant tumor-specific stem-like CD8^+^ T cells, including Ttsm and Tpex cells, which have substantial proliferative potential and provide a source of effector T cells that respond to immune checkpoint blockade (ICB) and continuously enter the TME [2]. During immune surveillance and tumor clearance, mature dendritic cells (DCs) that have captured tumor antigens migrate to TDLNs, cross-present antigens to naive and memory lymphocytes, and drive clonal expansion and effector differentiation [3,4,5].

As tumors progress, TDLNs can also be remodeled into premetastatic niches. Before cancer cells colonize these sites, factors released by the primary tumor can induce substantial structural and immunological remodeling in TDLNs, including enhanced lymphangiogenesis, dedifferentiation of high endothelial venules (HEVs), and disruption of fibroblastic reticular cells (FRCs) [6,7,8,9]. At the same time, the immune landscape of TDLNs is reshaped, with impaired DC maturation, T-cell exhaustion, and abnormal accumulation of immunosuppressive populations such as regulatory T cells (Tregs) and myeloid-derived suppressor cells (MDSCs) [5,10,11]. TDLNs therefore embody a central immunological tension: immune activation and immunosuppression coexist within the same organ, yet the molecular and spatial basis of this tension remains incompletely understood.

Flow cytometry and bulk RNA-seq have contributed substantially to previous studies of TDLNs, but their limitations become increasingly evident when they are applied to the cellular heterogeneity and microenvironmental topology of these organs. Tissue dissociation during flow cytometry inevitably removes in situ spatial information and physical cell–cell interaction networks. In addition, limited measurement dimensions make it difficult to identify rare or transitional cell subsets in an unbiased manner [12,13]. Bulk RNA-seq, by contrast, measures average gene expression across tissue. This signal-dilution effect can mask dynamic changes in small populations of tumor-specific immune cells against a large background of resting lymphocytes. As a result, single-cell resolution is lost, spatial relationships among cells cannot be reconstructed, and it remains difficult to determine where activation and suppressive signals arise within TDLNs [14]. These limitations have driven a shift toward high-resolution single-cell and spatial multi-omics technologies that can better resolve the dynamic spatiotemporal remodeling of the tumor immune microenvironment.

Spatial multi-omics technologies, including spatial transcriptomics such as 10x Visium, Stereo-seq, and MERFISH, and spatial proteomics such as imaging mass cytometry (IMC), multiplexed ion beam imaging (MIBI), and CODEX, help address these limitations. These approaches preserve the ordered anatomical compartments of TDLNs while enabling direct analysis of physical cell–cell interactions. They also allow investigators to localize micrometastatic niches and extract high-dimensional markers based on spatial topology rather than cell proportions alone. Recent work has emphasized spatial mapping of TDLNs as an approach to studying systemic antitumor immunity and has supported the integration of spatial transcriptomics with multiplexed protein imaging [15]. This direction has begun to yield concrete findings. A recent Nature Communications study used Stereo-seq to generate a high-resolution spatial atlas of primary breast tumors and paired metastatic TDLNs, revealing the cellular and molecular architecture of venous niches associated with lymphocyte extravasation and illustrating the utility of this technology for resolving anatomical organization [16]. In a multi-omics study of head and neck cancer, spatial proteomics and transcriptomics identified an immunosuppressive fibroblast–myeloid niche induced by lymph node colonization. Spatial intersection of this niche with lymphoid follicles was associated with T-cell dysfunction and Treg activation, supporting the use of spatial technologies to capture immune-remodeling networks in situ [17]. Spatial atlases are also refining our understanding of mechanisms associated with immunotherapy response. A Nature Immunology study showed that TDLNs function as a distinct spatial niche that maintains KLF2-dependent effector CD8+ T-cell differentiation and supports the proliferative expansion and systemic dissemination of CX3CR1+ effector T cells during ICB therapy [18].

Taken together, current evidence supports an emerging conceptual shift: TDLNs are not immunologically homogeneous organs, but comprise multiple spatial compartments with distinct functions. Their spatial arrangement, particularly the relative positions and boundaries between immune activation regions and immunosuppressive regions, may influence whether TDLNs support antitumor immunity or facilitate immune escape. This review focuses on this developing area and addresses three questions: how spatial multi-omics technologies can resolve activation-suppression compartmentalization within TDLNs; what molecular mechanisms regulate the formation and maintenance of these spatial compartments; and how spatial immune coding in TDLNs may inform future strategies for precision immunotherapy.

## 2. Technical Toolbox for Decoding TDLN Spatial Organization

TDLNs are secondary lymphoid organs with highly organized architecture, comprising T-cell zones, B-cell follicles, germinal centers, the medulla, subcapsular sinuses, and high endothelial venules (HEVs). Tumor progression can alter immune-cell composition, stromal-cell phenotypes, and chemokine gradients within these compartments. These changes do not occur evenly across the entire lymph node; instead, they often emerge in specific regions and cellular neighborhoods. Consequently, approaches such as flow cytometry, bulk RNA sequencing, and conventional immunohistochemistry provide only a partial view because they cannot fully capture cellular identity, functional state, and spatial organization at the same time. Spatial omics addresses this limitation by linking molecular profiles and cell identities to precise tissue context, allowing lymph node architecture to be preserved while functional compartmentalization in TDLNs is analyzed.

### 2.1. Spatial Transcriptomics

Spatial transcriptomics is mainly used to define the in situ spatial distribution of gene expression. Depending on the technical workflow, current methods can be broadly divided into NGS-based spatial transcriptomics, which couples spatial barcoding with high-throughput sequencing, and image-based in situ transcriptomics. The former includes platforms such as 10x Genomics Visium, Slide-seq, and Stereo-seq. Its main advantage is relatively unbiased transcriptome-wide profiling, making it suitable for identifying new cell states, stromal-remodeling signals, and potential spatial niches across the scale of an entire lymph node [19,20,21,22,23,24,25,26]. In TDLN studies, these approaches are particularly useful for mapping the overall immune landscape and screening molecules associated with the decline of immune activation or the emergence of immunosuppression. By contrast, image-based spatial transcriptomic methods, such as MERFISH, seqFISH, STARmap, Xenium, and CosMx, emphasize in situ localization at single-cell or even subcellular resolution [20,27,28,29,30,31,32,33]. These methods usually require predefined gene panels and are therefore better suited to validating specific cell populations and their spatial relationships on the basis of prior evidence. For example, in studies of TDLN functional compartmentalization, image-based spatial transcriptomics can be used to assess whether Tregs, exhausted T cells, myeloid cells, CAFs, FRCs, and other cell populations form stable neighborhoods within specific anatomical regions. It can also help determine whether immunosuppressive niches spatially intersect with B-cell follicles, perifollicular T-cell zones, or HEV-associated regions.

### 2.2. Spatial Proteomics

Spatial proteomics detects multiple protein markers in situ and can more directly reflect cellular phenotypes and functional states. Highly multiplexed imaging platforms, including IMC, MIBI, and CODEX/PhenoCycler, enable immune-cell phenotyping, functional-state identification, and spatial neighborhood analysis at single-cell or subcellular resolution [34,35,36,37,38,39,40]. For TDLN studies, these methods can delineate structures such as T-cell zones, B-cell follicles, subcapsular sinuses, and the medulla with relatively high clarity, and can further characterize spatial features such as T-cell exhaustion, Treg activation, myeloid-cell enrichment, and CAF-associated suppressive niches.

In addition to highly multiplexed imaging technologies, region-of-interest-based spatial profiling approaches such as GeoMx DSP can compare transcriptional or protein-expression differences across distinct tissue regions. They are suitable for examining molecular differences among areas adjacent to metastatic foci, relatively preserved distal regions, and tumor-negative lymph nodes [41,42,43,44]. Antibody-independent techniques, including MALDI-MSI and deep visual proteomics, can complement conventional spatial immune profiling at the levels of proteins, peptides, lipids, metabolites, and post-translational modifications, and may help identify molecular markers involved in TDLN remodeling [45,46,47,48].

### 2.3. Multi-Omics Integration Strategies

Because TDLN remodeling involves changes in immune-cell states, restructuring of stromal networks, alterations in blood and lymphatic vessels, and local cell–cell interactions, a single spatial technology is often insufficient to fully explain changes in functional compartmentalization. A reasonable workflow is to use NGS-based spatial transcriptomics for global, unbiased screening, followed by validation of candidate cell populations and their spatial proximity at single-cell resolution using image-based spatial transcriptomics or spatial proteomics. When combined with TCR/BCR clonal tracking, cell–cell communication analysis, and spatial neighborhood analysis, these approaches can help determine whether antigen-specific lymphocytes are effectively activated and expanded in TDLNs or gradually shift toward exhaustion under the influence of local suppressive niches [33,35,49,50,51]. Thus, spatial multi-omics provides more than higher-dimensional data; it offers a technical framework for analyzing the formation, evolution, and function of immune activation regions and immunosuppressive regions within TDLNs. The technical features of these approaches and their applicable scenarios in TDLN research are summarized in Table 1.

### 2.4. Current Limitations and Future Directions of Spatial Profiling Technologies

Most current spatial studies of TDLNs are performed using two-dimensional tissue sections, which provide valuable information regarding cellular composition and local spatial relationships but may not fully capture the three-dimensional organization of lymph nodes. TDLNs contain highly structured microanatomical compartments, including lymphatic sinuses, high endothelial venules, T-cell zones, and B-cell follicles, whose spatial relationships extend across multiple tissue planes. Consequently, two-dimensional analyses may underestimate the continuity and spatial heterogeneity of immune niches [52]. Three-dimensional imaging and volumetric reconstruction approaches, such as light-sheet microscopy and serial-section reconstruction, may provide additional insights into the organization of immune niches, lymphatic remodeling, and tumor–immune interactions within TDLNs.

Second, although spatial transcriptomic and spatial proteomic approaches provide complementary information, their integration remains technically challenging. Spatial transcriptomics enables the identification of spatially resolved molecular programs, whereas spatial proteomic imaging directly characterizes protein-defined cellular phenotypes and spatial relationships. Recent studies have demonstrated the value of combining these approaches in TDLNs. For example, Haist et al. integrated CODEX-based spatial proteomic imaging with spatial transcriptomic validation to identify fibroblast–myeloid immunosuppressive niches in metastatic lymph nodes, revealing spatially organized stromal and immune compartments associated with immune dysfunction [17]. Beyond TDLN studies, emerging spatial multi-omic approaches have demonstrated the feasibility of integrating transcriptomic and proteomic information within the same tissue context, providing opportunities to link molecular programs with protein-defined cellular phenotypes [53]. However, such approaches remain technically challenging and have not yet been widely applied to TDLN research, with current studies mainly relying on complementary profiling strategies, such as matched samples or serial tissue sections, rather than simultaneous RNA and protein detection within the same tissue section. Future multimodal spatial technologies may further connect transcriptional programs with functional protein states and improve the characterization of TDLN immune niches.

Third, spatial profiling technologies generate large and complex datasets, and differences in tissue preparation, imaging platforms, marker panels, and computational pipelines may influence the identification and interpretation of spatial niches. Although advanced algorithms have facilitated cell segmentation, spatial neighborhood analysis, and cell–cell interaction inference, standardized analytical frameworks and cross-platform validation remain lacking. Establishing unified criteria for defining immune niches will be important for comparing findings across studies and translating spatial features into reproducible biomarkers.

Finally, the clinical application of spatial profiling in TDLNs remains limited by tissue availability, cost, and the need for robust validation in large patient cohorts. Many spatial studies are currently exploratory and performed in relatively small cohorts, making it difficult to determine whether identified spatial features can serve as clinically useful biomarkers for prognosis prediction or treatment selection. Future studies integrating spatial profiling with longitudinal clinical data, functional assays, and therapeutic response information will be required to facilitate clinical translation.

## 3. Spatial Atlas of TDLN Functional Compartmentalization

As typical secondary lymphoid organs, TDLNs comprise multiple anatomically distinct but functionally coordinated compartments, including B-cell follicles with germinal centers, T-cell zones or paracortical regions, the medulla, and subcapsular sinuses. These compartments support specialized immune functions: B cells undergo clonal expansion and somatic hypermutation in the dark zone of germinal centers and are selected for antigen recognition and affinity maturation in the light zone; T cells are activated and differentiate in the paracortex after interaction with DCs; and plasma cells and memory cells accumulate in the medulla and support antibody output [54]. Tumor involvement, however, can reshape the immune properties of these regions. Active immune foci and suppressive foci may coexist within the same anatomical compartment, and different anatomical regions may be connected through functional links to form integrated immunosuppressive networks or immune activation chains [55].

Advances in spatial omics have enabled investigators to examine the geometric distribution of immune effector states within TDLNs at high resolution. Studies using spatial transcriptomics, spatial proteomics, and multiplexed imaging indicate that, during the dynamic interaction between tumor development and immune escape, TDLNs should no longer be viewed as homogeneous anatomical organs. Instead, they can be functionally partitioned into relatively segregated local microenvironments [56,], or niches: immune activation niches dominated by effector-cell expansion and active antigen presentation [57], and immunosuppressive niches formed by aggregation of Tregs, suppressive myeloid cells, and immune-checkpoint networks [17]. Spatial omics therefore adds molecular resolution to classical anatomical compartmentalization.

### 3.1. Tumor-Driven Early Cellular and Spatial Remodeling in TDLNs

Spatial omics studies indicate that tumor-induced TDLN remodeling can begin before overt metastasis and becomes more pronounced after tumor colonization. This process may develop gradually under the sustained release of soluble factors, inflammatory mediators, and extracellular vesicles by tumors. Under physiological conditions, FRCs, FDCs, lymphatic endothelial cells (LECs), and HEVs jointly establish chemokine gradients that maintain the stable organization of T-cell zones, B-cell follicles, and germinal centers. Tumors, however, can act on stromal and immune cells in TDLNs through distant signals and alter their spatial organization. In murine models of triple-negative breast cancer, Mattavelli et al. integrated 10x Genomics Visium spatial transcriptomics with immunofluorescence imaging and an scRNA-seq-derived reference atlas, followed by cell2location-based spatial deconvolution. This approach that triple-negative breast cancer can reprogram FRCs through TLR4 signaling, inducing CCL2 and CCL7 secretion, recruitment of immunosuppressive monocytes, and formation of suppressive cellular clusters around HEVs [58]. Extracellular vesicles derived from head and neck squamous cell carcinoma can activate the JAK1-STAT1 pathway in FRCs, upregulate PD-L1 expression, induce CD8+ T-cell exhaustion, and promote formation of a metastasis-associated microenvironment [59]. In addition to fibroblastic reticular cells, lymphatic endothelial cells—key components of the lymph node stromal network—are also regulated by tumor-derived signals. Eichin et al. paired metastatic and non-metastatic sentinel lymph nodes from treatment-naive breast cancer patients and performed combined profiling using 10x Genomics Chromium Single Cell 3′ RNA-seq and high-resolution 10x Genomics Visium HD spatial transcriptomics, with sequencing carried out on the Illumina NovaSeq X platform. Space Ranger HD and Seurat software were utilized for spatial data processing and integrative analysis. Their results revealed profound remodeling of LEC subpopulations upon lymph node metastasis: immunoregulatory capsular sinus LECs and medullary sinus LECs were depleted, whereas paracortical sinus-associated LECs underwent aberrant expansion, accompanied by upregulated expression of molecules such as matrix Gla protein (MGP). Follow-up experiments further demonstrated that elevated MGP facilitates adhesion between tumor cells and lymphatic endothelium. Collectively, these findings indicate that tumor metastasis remodels not only lymph node immune cell populations and fibroblastic stromal networks but also that the lymphatic endothelial system actively contributes to the construction of a metastasis-associated microenvironment [60].

In recent years, studies have employed NanoString nCounter PanCancer Immune Profiling/Cancer Pathways panels in combination with Opal multiplex immunofluorescence, along with Vectra 3 imaging and spatial quantitative analyses using inForm and QuPath. These investigations have found that, in addition to stromal cells, the composition and functional phenotypes of immune cells within tumor-draining lymph nodes of triple-negative breast cancer also undergo profound alterations. Spatial proteomic analysis further revealed that IL4^+^ mast cells are predominantly enriched in the lymphatic sinuses and T-cell zones. The IL-4 secreted by these cells acts on adjacent dendritic cells and helper T cells, driving Th2 polarization and ultimately impairing antitumor immune responses [61]. In melanoma-related studies, cyclic multiplex immunofluorescence imaging has further revealed that tumor-derived extracellular vesicles can induce cells to express CD36, thereby promoting the accumulation of myeloid-derived suppressor cells. CD36^+^ cells were found to be highly co-localized with CD209^+^ tolerogenic dendritic cells and CD163^+^ M2-like macrophages, yet exhibited significantly increased spatial distance from CD4^+^ and CD8^+^ T cells, collectively establishing a myeloid-cell-dominated immunosuppressive microenvironment [62].

During the continuous remodeling of stromal cells and immune cells, the chemokine network that maintains the normal compartmental architecture of lymph nodes gradually disintegrates. Czernilofsky et al. using BD Rhapsody single-cell full-length transcriptomics combined with 10x Genomics Xenium Prime 5K imaging-based spatial transcriptomics, found that tumor-associated inflammatory signals disrupt the spatial concentration gradients established by chemokines such as CXCL13, CCL19, and CCL21, thereby impairing cell migration and compartmentalization mechanisms, and ultimately leading to the progressive collapse of the normal spatial architecture of lymph nodes [63]. Thus, early TDLN remodeling is not simply a change in a single cell population. Rather, it is a systemic spatial remodeling process in which stromal-cell reprogramming disrupts chemokine networks and reshapes immune-cell composition, localization, and interactions, thereby laying the groundwork for subsequent decline of immune activation regions and formation of immunosuppressive niches.

### 3.2. Decline of Immune Activation Regions

In normal LNs, the paracortex, B-cell follicles, and germinal centers together form the core functional regions for immune activation, supporting antigen presentation, T-cell priming, and B-cell maturation [64]. The paracortex is the main site of interaction between DCs and naive T cells and is responsible for inducing antigen-specific T-cell responses. B-cell follicles and germinal centers mediate B-cell proliferation, affinity maturation, and effector differentiation, thereby supporting humoral immunity [65]. These functional regions maintain lymph node homeostasis through coordinated cell migration and intercellular communication, forming an efficient antitumor immune microenvironment. However, under continuous tumor drainage and tumor-associated immunosuppressive signals, the stromal networks, chemokine gradients, and immune-cell interactions that maintain these regions gradually change, leading to a decline in immune activation.

First, the structural basis that sustains immune activation regions changes substantially. High endothelial venules (HEVs) are specialized postcapillary venules located in the paracortex of lymph nodes. Their endothelial cells express peripheral node addressin (PNAd) and multiple chemokines, making them the main gateway through which circulating lymphocytes enter lymph nodes. By mediating lymphocyte rolling, adhesion, and transendothelial migration, HEVs continuously recruit naive T cells, B cells, and subsets of memory lymphocytes into lymph nodes and guide them to appropriate functional regions, thereby maintaining immune surveillance and initiating adaptive immune responses []. In normal TDLNs, FRCs, FDCs, and endothelial cells jointly construct chemokine networks. FRCs mainly secrete CCL19 and CCL21 to recruit CCR7+ T cells and DCs to the paracortex, whereas FDCs attract CXCR5+ B cells and follicular helper T cells to follicles and germinal centers through CXCL13. These processes establish clear spatial separation between T-cell and B-cell zones and support efficient immune-cell interactions [66,67]. Spatial analysis comparing nonmetastatic TDLNs with metastatic TDLNs (TDLN+) showed that TDLN+ contained fewer HEVs, collapsed vascular lumens, and reduced PNAd expression. FDCs (CD21+) and conventional DCs (CD11c+) were also significantly decreased, consistent with impaired lymphocyte recruitment, antigen presentation, and T-cell activation. CD8^+^ T cells were largely restricted to areas surrounding metastatic lesions and showed limited infiltration into the tumor core [68]. In parallel, tumor-associated inflammatory signals can reprogram FRCs, FDCs, and endothelial cells through IFN-gamma feedback loops, disrupting spatial gradients established by CXCL13, CXCL12, CCL19, and CCL21 and causing progressive blurring of B-cell/T-cell boundaries and follicular disorganization [59].

On this basis, T-cell functional states are further altered. Llaó-Cid et al., using a combined approach of 42-marker Helios mass cytometry, 10x Genomics Chromium Next GEM 5′ single-cell RNA/TCR sequencing, and 15-color RareCyte Orion multiplex immunofluorescence imaging, demonstrated that following tumor invasion of lymph nodes, the normal compartmental architecture of T and B cells gradually disappears; precursor exhausted T cells continuously differentiate into terminally exhausted T cells; and CD4^+^ regulatory T cells exhibit high spatial co-localization with PD-1^+^CD8^+^ T cells, collectively forming a locally immunosuppressive microenvironment [69]. These findings suggest that tumors not only impair T-cell recruitment and activation but also promote effector T-cell exhaustion.

Beyond the T-cell zone, B-cell follicles and germinal centers are also substantially affected. Disruption of the chemokine network reduces follicular integrity and weakens B-cell maturation and humoral immune responses. By applying first-generation barcode-array-based spatial transcriptomics and 10x Genomics Chromium Single Cell 3′ single-cell RNA sequencing, spatial transcriptomic studies in esophageal squamous cell carcinoma and stage III melanoma have further revealed that even within the same tumor-draining lymph node, regions distant from metastatic foci retain normal follicular and T-cell zone architectures, with enrichment of naive T cells, normal B cells, and antigen-presenting cells; whereas areas adjacent to metastatic lesions progressively lose these features. This suggests that immune-activated regions do not disappear completely, but instead continuously shrink and become marginalized under persistent tumor stimulation [70,71].

This spatial remodeling process has also been observed in non-small-cell lung cancer-associated lymph nodes. Spatial multiplex immunohistochemistry showed that immune cells in nonmetastatic lymph nodes (MNLNs) were widely distributed in the paracortex and displayed an immune-activated state. In metastatic lymph nodes (MPLNs), however, immune cells gradually accumulated at the invasive tumor margin, whereas immune cells within tumor regions were markedly reduced. These findings indicate that formerly continuous immune activation regions become compressed and marginalized [72].

In summary, spatial technologies show that immune activation regions in TDLNs do not vanish abruptly. Rather, under sustained tumor pressure, they undergo a dynamic process involving disruption of structural networks, imbalance of immune-cell function, and contraction of spatial extent. This process provides a basis for later formation of immunosuppressive niches and establishment of an immune activation-immunosuppressive spatial pattern.

### 3.3. Establishment of Immunosuppressive Regions

Immunosuppressive cell populations and immunosuppressive microenvironments have long been recognized in conventional immunology. However, their spatial organization within TDLNs and their relationship to immune activation regions have not been systematically resolved. With the development of spatial transcriptomics, spatial proteomics, and multiplexed imaging, researchers can now analyze the spatial localization, cellular interactions, and niche architecture of immunosuppressive cells in situ. These approaches reveal the spatial basis for the formation and expansion of immunosuppressive regions in TDLNs. In parallel with the gradual decline of immune activation regions, new spatial niches jointly constructed by immunosuppressive cells and stromal cells begin to emerge within TDLNs. Spatial omics studies indicate that these suppressive cells are not randomly distributed, but instead aggregate into local immunosuppressive regions.

In models of triple-negative breast cancer, integrated spatial and single-cell transcriptomic analyses showed that monocytes and FRCs were mainly distributed in T-cell regions, the medulla, and areas surrounding HEVs, where they formed highly colocalized structures. Through TLR4 signaling, FRCs upregulated CCL2 and CCL7, continuously recruited immunosuppressive monocytes with high PD-L1 and iNOS expression, and formed an FRC–monocyte immunosuppressive niche centered on mMDSC-like cells, thereby promoting T-cell exhaustion and distant metastasis [58]. Another study of triple-negative breast cancer using spatial proteomics found that large numbers of IL4^+^ mast cells were enriched in lymph node sinuses and T-cell regions. Their secreted IL-4 acted on neighboring DCs and helper T cells and induced naive CD4^+^ T cells to polarize toward Th2 cells, thereby forming a local microenvironment favorable to immunosuppression [61]. Melanoma studies showed that tumor-derived extracellular vesicles induced CD36 expression and promoted aggregation of myeloid suppressor cells. Spatial colocalization analysis found that CD36+ cells were highly adjacent to CD209+ tolerogenic DCs and CD163+ M2-like macrophages, but were clearly separated from CD4^+^ and CD8^+^ T cells, forming a myeloid-cell-enriched and T-cell-excluded architecture [62]

As metastasis progresses, suppressive niches become more complex. Using Singleron GEXSCOPE single-cell RNA sequencing combined with 10x Genomics Visium spatial transcriptomics, it was revealed that in metastatic lymph nodes of breast cancer, macrophages gradually shift toward a pro-tumor phenotype characterized by high expression of CD163 and SPP1, and form cellular aggregates around tumor cells. Meanwhile, osteoclast-like giant cells are closely adjacent to tumor cells, and SPP1 promotes tumor invasion and metastasis through short-range paracrine signaling [73]. This observation suggests that suppressive niches may not only weaken antitumor immune responses but also contribute to metastasis-associated microenvironments.

Stromal cells also participate in the construction of suppressive regions. In metastatic lymph nodes from intrahepatic cholangiocarcinoma, THBS1^+^ myCAFs expand markedly and remodel the collagen matrix and lymphatic network, forming highly suppressive spatial units together with CD36^+^ macrophages, Tex cells, and Tregs [74]; Using 10x Genomics single-cell RNA sequencing and 10x Genomics Visium spatial transcriptomics technology, osteosarcoma studies have also identified stable colocalization between CAFs and SPP1^+^ macrophages [75].

By means of CODEX/PhenoCycler Fusion high-throughput spatial protein imaging and Akoya imaging-based spatial transcriptomics, Haist et al. described a myeloid–CAF niche in metastatic TDLNs, characterized by spatially organized cellular neighborhoods containing CAFs, macrophages, and plasma cells. These niches were associated with persistent activation of IFN-gamma-, TGF-beta1-, and CXCL10-related signaling programs, indicating a locally immunosuppressive and stromal-remodeled microenvironment. Similar myeloid–CAF niches were detected not only within tumor-involved regions, but also in adjacent uninvolved areas and ipsilateral tumor-negative lymph nodes. This finding suggests that suppressive spatial remodeling in TDLNs may not be restricted to overt metastatic foci, but can extend to anatomically connected lymph node regions [17].

Together, spatial technologies suggest a core mechanism underlying the formation of immunosuppressive regions: tumors remodel stromal-cell and myeloid-cell networks to establish suppressive spatial units, including FRC–monocyte niches, CD36^+^ myeloid niches, and myeloid–CAF niches, which may then extend across the TDLN system.

### 3.4. Spatial Pattern of Immune Activation and Immunosuppressive Regions

As immune activation regions decline and immunosuppressive regions expand, TDLNs gradually shift from relatively homogeneous immune organs into functional systems with marked spatial heterogeneity. Spatial omics studies indicate that tumor-driven TDLN remodeling is not limited to functional decline of immune activation regions or formation of immunosuppressive niches. It also reshapes the spatial relationships among different functional regions.

Studies of esophageal squamous cell carcinoma, melanoma, intrahepatic cholangiocarcinoma, and osteosarcoma consistently show that regions distant from metastatic foci can retain normal follicular and T-cell-zone structures and remain enriched for naive T cells, normal B cells, and antigen-presenting cells. By contrast, regions adjacent to metastatic foci are enriched for suppressive populations, including Tregs, exhausted T cells, TREM2^+^ macrophages, CAFs, and SPP1^+^ myeloid cells [70,71,74,75]. Together, these studies indicate that immune activation and immunosuppression are not evenly distributed throughout the lymph node, but form functionally distinct regions with clear spatial heterogeneity at the tissue level.

Haist et al. further clarified the mechanism by which this spatial coding may emerge. Under normal conditions, the paracortical T-cell zone (PFTZ) and lymphoid nodular follicle (LNF), which support T-cell priming and B-cell activation, are closely associated with networks of T cells, B cells, and DCs. In metastatic TDLNs, however, myeloid–CAF niches progressively intersect spatially with these immune activation regions. As this spatial intersection increases, Treg activation, T-cell exhaustion, and accumulation of PD-L1-high myeloid cells appear within the PFTZ and LNF. This pattern suggests that formerly separate immune activation and immunosuppressive regions become spatially coupled, allowing suppressive signals to influence the functional state of adjacent activation regions through local cell–cell interactions [17].

**Figure 2:**
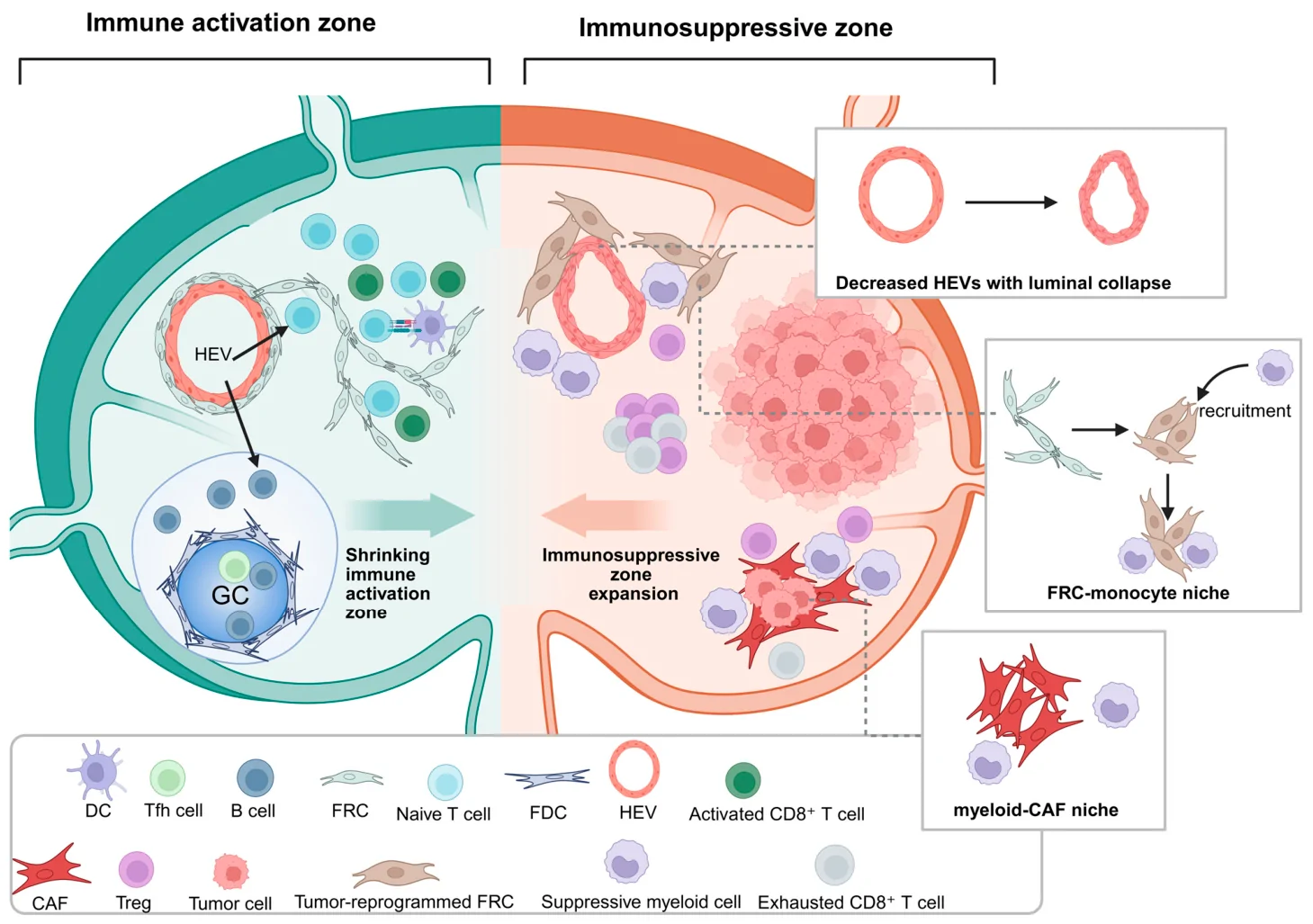
Spatial remodeling of immune activation and immunosuppressive zones in TDLNs. Tumor-driven remodeling of TDLNs can lead to contraction of the immune-activation zone and expansion of the immunosuppressive zone. The activation zone contains relatively preserved HEVs, FRC networks, DC–T-cell interactions, activated CD8^+^ T cells, and B-cell follicle/germinal-center structures. In contrast, the immunosuppressive zone is enriched in tumor cells, tumor-reprogrammed FRCs, suppressive myeloid cells, Tregs, exhausted CD8^+^ T cells, and CAF-associated niches, accompanied by impaired HEVs, FRC–monocyte niches, and myeloid–CAF niches. This schematic represents a conceptual model rather than a universal structure present in all TDLNs.

**Figure 3:**
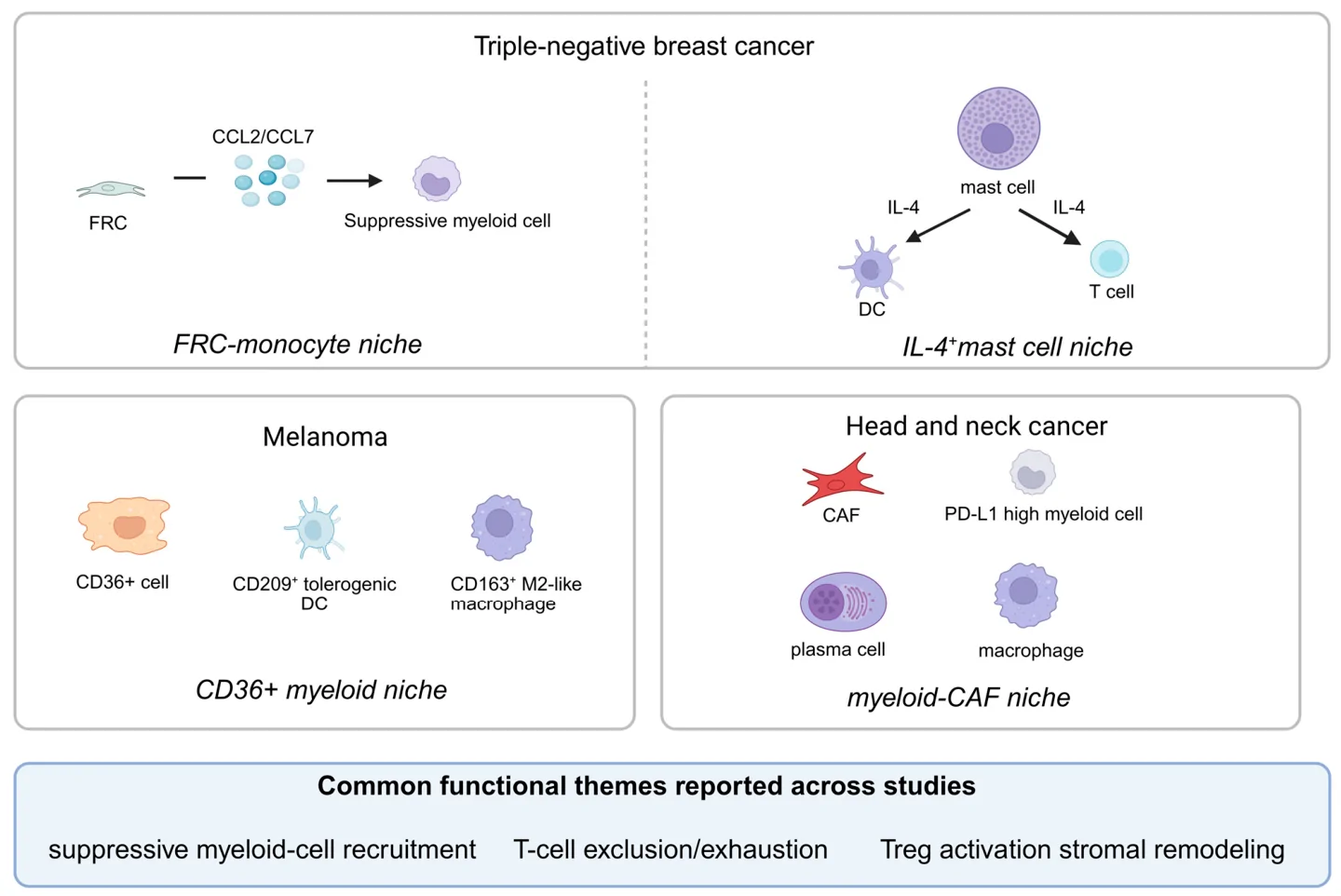
Representative immunosuppressive niches reported across cancer-associated TDLNs. Spatial studies have identified diverse stromal–immune cellular neighborhoods in TDLNs, including FRC–monocyte and IL-4^+^ mast cell niches in triple-negative breast cancer, CD36^+^ myeloid niches in melanoma, and myeloid–CAF niches in head and neck cancer. Although these niches differ in their cellular composition and spatial organization, they converge on common immunosuppressive features, including myeloid-cell recruitment, T-cell dysfunction/exhaustion, Treg activation, and stromal remodeling. These examples represent tumor-specific spatial programs and should not be considered universal features of all TDLNs.

Thus, spatial technologies show that immune escape in TDLNs does not arise only from a decrease in immune-activating cells or an increase in immunosuppressive cells. It also reflects changes in the spatial relationships among functional regions. As immune activation regions become increasingly restricted to areas distant from metastatic foci, while immunosuppressive regions expand around tumors and become spatially coupled with activation regions, TDLNs may acquire a spatial coding pattern characterized by the coexistence of immune activation regions and immunosuppressive regions. Within this pattern, cellular functional states are shaped not only by intrinsic molecular features but also by spatial location and neighboring niches. Collectively, these findings suggest that tumor-driven TDLN remodeling is not merely reflected by changes in immune-cell abundance, but by the spatial redistribution of functionally distinct immune regions. Relatively preserved immune-activation regions, characterized by HEV-associated lymphocyte entry, DC–T-cell priming, and B-cell follicle/germinal-center activity, may progressively shrink, whereas immunosuppressive regions enriched in tumor-reprogrammed stromal cells, suppressive myeloid cells, Tregs, exhausted CD8^+^ T cells, and myeloid–CAF or FRC–monocyte niches expand around metastatic or tumor-conditioned areas. This spatial imbalance is summarized in Figure 2. Across different cancer types, spatial profiling studies have identified diverse immunosuppressive niches within TDLNs, characterized by distinct combinations of stromal and immune-cell populations. Representative examples, including FRC–monocyte, IL-4^+^ mast-cell, CD36^+^ myeloid, and myeloid–CAF niches, are summarized in Figure 3.

## 4. Implications of Spatially Resolved TLS Functional Compartmentalization for TDLN Research

Although spatial transcriptomics, spatial proteomics, and multiplexed imaging have substantially advanced TDLN research in recent years, studies that use spatial technologies to resolve TDLN functional compartmentalization remain relatively limited, and current evidence comes mainly from spatial omics studies in a small number of tumor types. By contrast, spatial technologies have been applied more extensively in studies of tumor tertiary lymphoid structures (TLSs). Researchers have used spatial omics to systematically characterize the organization of functional regions within TLSs, including T-cell zones, B-cell follicles, germinal center-like structures, and HEVs, and to relate these regions to antitumor immune responses. Mature TLSs and normal lymph nodes share important organizational features, including highly organized immune microenvironments composed of T-cell zones, B-cell follicles, DC networks, and HEVs [76]. The spatial framework developed in TLS research therefore provides a useful reference for spatial analysis of TDLN functional compartmentalization, while also helping frame local antitumor immune responses. Although TLSs and TDLNs occupy different anatomical contexts and perform different biological functions, TLS studies have established approaches for identifying functional compartments, resolving spatial niches, and reconstructing tissue evolution trajectories. These approaches can inform future studies of spatial coding mechanisms in TDLNs.

### 4.1. From Cellular Composition to Functional Compartments: The TLS Spatial Atlas Paradigm

Spatial technologies have shifted TLS research from traditional descriptions of cellular composition toward analysis of functional compartmentalization. Pan-cancer spatial atlas studies of TLSs show that TLSs can progress from lymphocyte aggregates to primary follicle-like TLSs and secondary follicle-like TLSs, forming distinct spatial niches enriched for T/B cells, FDCs, endothelial cells, plasma cells, and mesenchymal cells [77]. Studies of TLSs in gastric cancer, nasopharyngeal carcinoma, and ovarian cancer also show that TLSs at different spatial locations or maturation states differ substantially in T cells, B cells, DCs, HEVs, and chemokine expression. Their immune activity therefore cannot be inferred simply from the presence or absence of TLSs [78,79,80]. These advances in TLS research suggest that future spatial studies of TDLNs should move beyond macroscopic anatomical assessment of whether the paracortex or follicular structures remain intact. Highly multiplexed in situ spatial protein imaging platforms, such as CODEX or CosMx, can preserve lymph node geometry while enabling fine cell phenotyping. In particular, deeper in situ mapping of paracortical T-cell zones (PFTZs) that appear morphologically normal and have not yet been infiltrated by tumor will be important for defining the functional boundaries of TDLNs under distant tumor regulation.

### 4.2. From Cell–Cell Interactions to Spatial Niches: Organizational Basis of TLS Functional Regions

TLS functional compartmentalization does not depend only on immune-cell aggregation. It is regulated by spatial niches composed of stromal cells, vascular endothelial cells, DCs, and lymphocytes. Studies have found that CCL19+ fibroblasts or CCL19^+^ perivascular cells can recruit T and B cells through chemokine networks and promote TLS formation [77,81]. IFN-responsive HEVs can recruit CXCR3^+^ CD4^+^ T cells through the CXCL9-CXCR3 axis and participate in TLS organization [78]. By contrast, IGLL5^+^ B cells can impair HEV function and disrupt TLS structure [82]. In addition, CD4+ Texprog/Tfh cells, germinal center B cells, DC-LAMP^+^ DCs, and LGALS2^+^ DCs within mature TLSs jointly participate in maintaining T-cell and B-cell responses [83,84]. These findings suggest that the formation and decline of TDLN functional regions may likewise be regulated by spatial interactions among FRCs, FDCs, HEVs, DCs, and lymphocytes. Future studies should focus on how chemokine networks, vascular niches, and antigen-presentation niches cooperate to maintain immune activation regions within TDLNs.

### 4.3. From Static Maps to Dynamic Evolution: Lessons for Studying TDLN Spatial Remodeling

Spatial technologies have also extended TLS research from static structural observation to dynamic evolutionary analysis. Studies of TLSs in ovarian cancer show that lymphoid structures can form a continuous maturation spectrum from lymphocyte aggregates to TLSs without germinal centers and then to TLSs containing germinal centers. As maturation increases, B-cell activation, immunoglobulin production, BCR signaling, and germinal center reactions are progressively enhanced [79]. Tang et al. further found that immature TLSs are not all at the same developmental stage. They can be divided into conforming TLSs with maturation potential and deviating TLSs with altered development. Tumor-derived tryptophan metabolism can interfere with B-cell differentiation into germinal center B cells, causing TLSs to deviate from normal maturation trajectories [85]. These studies suggest that TDLNs may also undergo a continuous transition from immune activation to structural remodeling and functional decline under persistent tumor stimulation. Future work could integrate spatial transcriptomics, spatial proteomics, cell-development trajectories, and cell–cell communication analysis to reconstruct spatial remodeling trajectories of different TDLN functional regions, thereby clarifying the mechanisms by which TDLNs shift from sites of antitumor immune priming to immunosuppressive microenvironments. Given the limited number of spatial-omics studies directly focusing on TDLN functional compartmentalization, spatial studies of tertiary lymphoid structures (TLSs) may provide a useful conceptual and methodological reference. TLSs and TDLNs share several organized lymphoid features, including T-cell areas, B-cell follicles, FDC networks, germinal-center-like reactions, and HEV-related regions. Therefore, findings from TLS spatial studies may help guide the definition of functional compartments, selection of spatial markers, and design of validation metrics in future TDLN spatial-omics research. However, because TLSs and TDLNs differ in anatomical location, developmental origin, and immune function, these findings should be interpreted as a reference framework rather than direct evidence for TDLN biology. The main implications are summarized in Table 2.

## 5. Clinical Implications and Perspectives

Spatial multi-omics analysis of TDLN functional compartmentalization offers a clinically relevant perspective for cancer immunotherapy and metastasis risk assessment. In conventional clinical practice, TDLNs are mainly viewed as anatomical indicators for tumor staging and metastatic assessment. Spatial omics studies suggest, however, that TDLNs are not merely passive routes for cancer-cell spread; they are key immune organs that influence whether antitumor immunity can be effectively initiated and maintained [86]. The relative distribution, spatial proximity, and dynamic changes of immune activation regions and immunosuppressive regions within TDLNs may reflect their immune function more accurately than immune-cell abundance or lymph node metastasis status alone. Future clinical assessment of TDLNs may therefore need to move beyond the presence or absence of metastasis and consider spatial immune state, incorporating features such as HEV integrity, DC antigen-presentation status, T-cell exhaustion, B-cell follicle and germinal center activity, and myeloid–stromal suppressive niches into an integrated evaluation.

For immunotherapy, TDLN spatial atlases may provide biomarkers for patient stratification and response prediction. Previous studies indicate that TDLNs can serve as important reservoirs of stem-like or precursor exhausted CD8^+^ T cells and contribute to effector T-cell expansion and export after ICB therapy [5]. Preserving and activating immune activation regions within TDLNs may therefore be important for improving responses to immunotherapy. Conversely, the presence of FRC–monocyte niches, CD36^+^ myeloid-cell-enriched regions, myeloid–CAF niches, or regions with strong Treg/Tex colocalization may indicate that a TDLN has shifted from an immune-priming site to a spatial platform for immunosuppression and metastatic tolerance. If validated, spatial risk scores based on these features could help identify patients likely to benefit from immunotherapy and provide a rationale for designing combination treatment strategies.

Mechanisms of TDLN spatial remodeling also suggest potential intervention strategies. On the one hand, immune activation regions might be reconstructed by restoring HEV function, maintaining FRC/FDC chemokine networks, enhancing DC antigen presentation, and improving T-cell priming. On the other hand, suppressive niches such as FRC–monocyte niches, IL4+ mast-cell niches, CD36+ myeloid niches, and myeloid–CAF niches could be targeted to reduce T-cell exhaustion and Treg activation. TLS studies further suggest that inducing organized lymphoid structures and functional antigen-presentation niches may help reshape immunosuppressive lymph node microenvironments toward immune activation. However, TDLNs and TLSs differ in anatomical location, developmental origin, and immune function. Regulatory mechanisms identified in TLSs should therefore not be directly equated with those in TDLNs, but rather used as a reference framework for understanding the formation and remodeling of spatial functional regions in TDLNs.

Several important questions remain for future research. First, most current spatial omics studies of TDLNs are cross-sectional and cannot directly trace the continuous transition of the same lymph node from immune activation to immunosuppression. Future studies should combine longitudinal samples, paired pre- and post-treatment samples, and animal models to reconstruct the temporal trajectory of TDLN spatial remodeling. Second, TDLN remodeling patterns may differ substantially across cancer types, metastatic stages, and treatment contexts, highlighting the need for cross-cancer analyses, standardized sampling, and unified analytical frameworks. Finally, integration of spatial transcriptomics, spatial proteomics, TCR/BCR clonal tracking, metabolic imaging, and artificial intelligence-based pathology may help construct multidimensional spatial atlases of TDLNs [87,88,89]. As these technologies mature, TDLNs may become spatial immune platforms that inform immunotherapy decisions, predict treatment response, and guide development of new combination strategies, in addition to their established role in metastatic assessment.

## Figures and Tables

**Table 1:** Common technologies for TDLN spatial omics research and their suitable applications.

Technology Type	Representative Platforms	Spatial Resolution	Detection Targets	Main Advantages	Main Limitations	TDLN Questions Suited to Address	Representative References
Spatial transcriptomics based on spatial barcoding and high-throughput sequencing (NGS-based ST)	10x Genomics Visium; Slide-seq; MGI Stereo-seq	Visium: about 50 μm; Slide-seq/Stereo-seq: single-cell or even nanoscale subcellular level	In situ captured mRNA followed by whole-transcriptome sequencing	Enables unbiased whole-transcriptome profiling without predefined targets; useful for identifying unknown cell types, screening biomarkers, and building spatial expression maps	RNA diffusion during permeabilization and in situ capture; relatively low capture efficiency; limited precision for single-cell assignment	Mapping the global immune landscape of whole lymph nodes and discovering exhausted immune subsets or stromal remodeling signals	[19,20,21,22,23,24,25,26]
Imaging-based spatial transcriptomics	MERFISH; seqFISH; STARmap; 10x Genomics Xenium; NanoString CosMx	True single-cell and subcellular spatial resolution	In situ RNA molecules; localization and quantification of hundreds to thousands of transcripts	Does not require tissue dissociation, nucleic acid extraction, or in vitro amplification; high molecular sensitivity; resolves subcellular mRNA distribution	Difficult to achieve unbiased whole-transcriptome coverage at low cost; risk of targeted bias; optical crowding can occur during multiple rounds of high-resolution imaging	Resolving spatial invasion of lymphoid follicles and perifollicular T-cell zones by immunosuppressive niches, local T-cell exhaustion, and abnormal Treg activation	[20,27,28,29,30,31,32,33]
Dual-platform and spatial multi-omics integration	NGS-based spatial technologies plus Xenium/CosMx; high-resolution spatial transcriptomics plus CODEX; 10x Visium V(D)J workflow	Platform-dependent	Spatial transcripts, spatial proteins, and TCR/BCR clonal distributions	Creates a workflow from unbiased discovery to high-resolution validation; can test whether RNA-level chemokine expression is translated into protein concentration gradients	Transcriptomics alone may not fully reflect the true functional states of immune cells; other limitations are not explicitly stated	Tracing the spatial distribution and expansion trajectories of antigen-specific clones in TDLNs and identifying regions where they are activated or driven toward exhaustion	[33,35,49,50,51]
Highly multiplexed imaging mass cytometry and cyclic fluorescence protein imaging	IMC; MIBI; CODEX/PhenoCycler	IMC/MIBI: subcellular resolution; CODEX: single-cell resolution	Multiplex proteins labeled by metal-isotope-conjugated antibodies or oligonucleotide-barcoded antibodies	Avoids autofluorescence and spectral overlap; supports targeted analysis of tens to hundreds of proteins; enables cell phenotyping and spatial neighborhood analysis	Complex data analysis; constrained by antibody specificity and coverage; three-dimensional spatial reconstruction remains challenging	Delineating T-cell zones, B-cell follicles, and subcapsular sinus boundaries, and identifying immune remodeling patterns and fibroblast–myeloid niches	[34,35,36,37,38,39,40]
Spatial sequencing based on oligonucleotide release	GeoMx Digital Spatial Profiler (DSP)	Region-of-interest (ROI) level; otherwise not explicitly stated	Protein signals from antibodies tagged with photocleavable oligonucleotides	Combines in situ labeling with high-throughput quantification; can quantify hundreds of proteins across selected regions or whole tissue sections; supports large-scale biomarker discovery	Complex data analysis; constrained by antibody specificity and coverage; other limitations are not explicitly stated	Comparing regional protein expression in TDLNs and integrating spatial transcriptomics to interpret spatial links between gene expression, protein function, and signaling pathways	[41,42,43,44]
Antibody-independent mass spectrometry-based spatial omics	MALDI-MSI; deep visual proteomics (DVP)	Platform-dependent	Proteins, peptides, metabolites, lipids, exogenous drug molecules, and post-translational modifications (PTMs)	Label-free and unbiased in situ molecular detection; expands the depth of protein and PTM identification in defined spatial regions	Platform-dependent	Discovering new molecular features of tumor-influenced TDLN microenvironments, identifying potential early warning biomarkers, and understanding spatial regulation of immune-cell activation and signaling	[45,46,47,48]

**Table 2:** Implications of TLS Spatial Functional Compartment Studies for TDLN Spatial Omics Research.

TLS Research Theme	Representative Spatial Findings in TLS	Functional Compartment/Spatial Niche	Implications for TDLN Studies	Recommended Technologies	Recommended Spatial Metrics for TDLN Validation	Key References
TLS maturity and functional compartmentalization	TLSs can progress from lymphoid aggregates to primary/secondary follicle-like structures or TLS+GC; mature TLSs are often accompanied by IgG+ plasma cells, germinal-center reactions, and higher immune activity.	T/B-cell zones, B-cell follicles, CD21+/CR2+ FDC networks, germinal-center-like structures, PNAd+/MECA-79+ HEV regions.	These findings suggest that TDLN studies should not only record whether anatomical regions are present, but should further evaluate the maturity and functional state of follicles, paracortical areas, and vascular regions.	mIF/IHC, CODEX or MIBI, 10x Visium or Visium HD, Xenium/CosMx for targeted validation.	CD3, CD4, CD8, CD20, CD21/CR2, CD23, AICDA, BCL6, Ki67, CD138/IgG; B/T zoning index, FDC network area, HEV density.	[77,79,82,85]
Identification of T/B-cell, FDC, and HEV spatial niches	Spatial transcriptomics and multiplex imaging can identify T/B-enriched areas, FDC-centered regions, and HEV-edge niches; IFN-responsive HEVs are linked to CXCL9-CXCR3-associated recruitment of CD4+ T cells into TLSs.	B-cell follicle/FDC center, T-cell zone, CXCL9+ IFN-HEV, CXCR3+CD4+ T-cell neighborhood, and location-specific TLS niches such as TC-TLS/IM-TLS.	This provides a reference for using cell-distance, regional-boundary, and neighborhood analyses to reconstruct molecular borders of PFTZ, LNF, HEV, and SCS regions in TDLNs.	GeoMx DSP, 10x Visium/Visium HD, multiplex IF, CODEX/MIBI, Xenium or CosMx.	CD20-CD3 distance, CD21+ centrality, PNAd/MECA-79+ HEV density, proportion of CXCL9+MECA-79+ HEVs, distance from CXCR3+CD4+ T cells to HEVs, region-specific immune-cell density.	[77,78,79,80]
Stromal, endothelial, and chemokine networks regulating TLS formation	CCL19+ fibroblasts/F2_CAFs and CCL19+ perivascular cells are associated with T/B-cell recruitment and TLS formation; TLS-adjacent stroma is enriched for chemokines, whereas CA-MSC-like stroma can weaken B-cell function and FDC-like differentiation.	CCL19+ fibroblasts, CCL19+ perivascular cells, TLS-adjacent stroma, arterial/HEV-like endothelium, and CA-MSC distal stromal niches.	These findings may guide spatial validation of FRC/FDC, vascular endothelial, and chemokine networks in TDLNs; future studies can test whether these niches participate in the formation or decline of immune-activation regions.	Stereo-seq or Visium HD for discovery; Xenium/CosMx RNAscope-like panels and mIF for chemokine validation; scRNA-seq integration.	CCL19, CCL21, CXCL13, CXCL9, CCR7, CXCR5, PDGFRB, MYH11, COL1A1, ICAM1/VCAM1, NOTCH1, WT1/CD200; stromal-lymphocyte distance.	[77,79,81]
Antigen presentation, Tfh/GC B-cell, and DC networks sustaining local immune responses in TLSs	In mature TLSs, CD4+ Texprog/Tfh cells, GC B cells, cDC2s, and DC-LAMP+ mregDCs participate in B-cell differentiation into plasma cells and support CD8+ T-cell activation; LGALS2 is associated with TLS-related DC activation.	Tfh/GC B-cell interaction zones, DC-LAMP/LAMP3+ DC regions, cDC2 antigen-presentation niches, and CD138+IgG+ plasma-cell niches.	These findings suggest that the antigen-presentation-helper T-cell-GC-like B-cell axis can be considered a candidate feature of immune-activation regions in TDLNs, but it should be independently validated in TDLN specimens.	Paired scRNA-seq/scTCR-BCR-seq with spatial transcriptomics; CODEX/MIBI or high-plex mIF for protein-level validation.	LAMP3/DC-LAMP, CD11c, HLA-DRA, CD86, CD40, ICOSLG, IL12B, CXCL13-CXCR5, CD40LG-CD40, BCL6, AICDA, CD138/IgG; spatial DC-Tfh-GC B triads.	[81,83,84]
Dynamic trajectories from immature to mature TLSs, or toward developmental deviation	Spatial trajectory analysis distinguishes immature TLSs into conforming TLSs with maturation potential and deviating TLSs; tumor-derived tryptophan metabolism is associated with restricted TLS maturation.	Mature TLSs, conforming/deviating TLSs, naive B-to-GC B developmental trajectories, and tryptophan/TDO2/kynurenine metabolic niches.	These findings suggest that TDLN spatial remodeling may follow continuous trajectories; future studies can test whether immune activation, structural remodeling, and functional decline correspond to distinct spatial developmental states in TDLNs.	Near-single-cell spatial transcriptomics such as Stereo-seq/Visium HD; spatial pseudotime with scRNA-seq/snATAC-seq integration; multiplex metabolic IHC.	Spatial pseudotime, naive B-to-GC B trajectory, CR2/CD21, CD23, AICDA, CXCL13, TDO2, IDO1, kynurenine, tryptophan-metabolism score, HEV-related gene sets.	[77,79,85]
Immunosuppressive factors disrupting TLS structure or function	IGLL5+ B cells can impair HEV function and promote TLS disassembly through IGLL5-LTBR interaction; tryptophan metabolism and CA-MSC-like stroma are also associated with restricted TLS maturation or weakened TLS function.	IGLL5+ B-cell-HEV neighborhoods, dilated/dysfunctional HEVs, TDO2/kynurenine metabolic niches, WT1+CD200+ CA-MSC-like stroma, and Treg/exhausted T-cell neighborhoods.	These findings suggest that TDLN studies should examine whether suppressive niches invade or surround immune-activation regions, and validate whether HEV injury, metabolic suppression, and stromal remodeling are associated with TDLN functional decline.	mIF/3D IF for HEV morphology, CODEX/MIBI for suppressive niches, spatial transcriptomics plus targeted metabolic staining.	IGLL5, LTBR, RELB/NFKB2, PNAd/MECA-79 and HEV morphology, TDO2/IDO1/kynurenine, WT1, CD200, CD73/CD90/CD105, FOXP3, PD-1/TIM-3/LAG-3 spatial proximity.	[79,80,82,85]

Note: TLSs and TDLNs share structural similarities, including organized T-cell areas, B-cell follicles, FDC networks, and HEV-related regions. However, they differ in anatomical location, developmental origin, and immune function. Therefore, the contents of this table should be used mainly as a reference framework for study design and spatial-marker selection in TDLN functional-compartment research, rather than as direct evidence that these mechanisms have been proven in TDLNs.

## Data Availability

No new data were created or analyzed in this study. Data sharing is not applicable to this article.

## Abbreviations

TDLN	Tumor-draining lymph node
TME	Tumor microenvironment
TLS	Tertiary lymphoid structure
ICB	Immune checkpoint blockade
DC	Dendritic cell
cDC	Conventional dendritic cell
FRC	Fibroblastic reticular cell
FDC	Follicular dendritic cell
LEC	Lymphatic endothelial cell
HEV	High endothelial venule
PNAd	Peripheral node addressin
Treg	Regulatory T cell
MDSC	Myeloid-derived suppressor cell
mMDSC	Monocytic myeloid-derived suppressor cell
CAF	Cancer-associated fibroblast
myCAF	Myofibroblastic cancer-associated fibroblast
Tfh cell	Follicular helper T cell
Th2 cell	Type 2 helper T cell
Tpex/TPEX	Precursor exhausted T cell
Tex/TEX	Exhausted T cell
Ttsm cell	Tumor-specific stem-like memory T cell
GC	Germinal center
PFTZ	Paracortical T-cell zone
LNF	Lymphoid nodular follicle
SCS	Subcapsular sinus
RNA-seq	RNA sequencing
NGS	Next-generation sequencing
scRNA-seq	Single-cell RNA sequencing
TCR	T-cell receptor
BCR	B-cell receptor
scTCR/BCR-seq	Single-cell T-cell receptor/B-cell receptor sequencing
snATAC-seq	Single-nucleus assay for transposase-accessible chromatin sequencing
ST	Spatial transcriptomics
MERFISH	Multiplexed error-robust fluorescence in situ hybridization
seqFISH	Sequential fluorescence in situ hybridization
STARmap	Spatially resolved transcript amplicon readout mapping
IMC	Imaging mass cytometry
MIBI	Multiplexed ion beam imaging
CODEX	Co-detection by indexing
DSP	Digital spatial profiling
GeoMx DSP	GeoMx digital spatial profiling
MALDI-MSI	Matrix-assisted laser desorption/ionization mass spectrometry imaging
mIF	Multiplex immunofluorescence
IHC	Immunohistochemistry
ROI	Region of interest
IFN	Interferon
IL	Interleukin
JAK1	Janus kinase 1
STAT1	Signal transducer and activator of transcription 1
TLR4	Toll-like receptor 4
KLF2	Krüppel-like factor 2
MGP	Matrix Gla protein
iNOS	Inducible nitric oxide synthase
PD-1	Programmed cell death protein 1
PD-L1	Programmed death-ligand 1
SPP1	Secreted phosphoprotein 1
THBS1	Thrombospondin 1
TNBC	Triple-negative breast cancer
NSCLC	Non-small-cell lung cancer
ESCC	Esophageal squamous cell carcinoma
iCCA	Intrahepatic cholangiocarcinoma
CLL	Chronic lymphocytic leukemia
HNSCC	Head and neck squamous cell carcinoma
CRLM	Colorectal cancer liver metastasis
